# BETTER TOGETHER: STRENTHENING THE AGE-FRIENDLY ECOSYSTEM

**DOI:** 10.1093/geroni/igad104.0332

**Published:** 2023-12-21

**Authors:** Holly Dabelko-Schoeny, Marisa Sheldon

**Affiliations:** The Ohio State University, Columbus, Ohio, United States; Ohio State University, Columbus, Ohio, United States

## Abstract

Frameworks to make communities, universities, public health systems, and health services more inclusive and equitable for older adults and people with disabilities have expanded over the last decade. Centering the voices of older adults, these “age-friendly” initiatives have sought social change through building knowledge, changing attitudes, and developing new practices and policies in a variety of settings. Despite the similarities in values and principles across these initiatives, these models of social change have largely operated independently from one another with unique administrative structures, networks, and resources. This presentation will utilize a macro application of the KAP (knowledge, attitude, practice) theory of behavior change to identify the vast opportunity for synergies across the age-friendly ecosystem, strengthening the potential for collective impact and lasting change. Often these initiatives have overlapping and intersecting goals and would benefit from intentional alignment across the initiatives. Modeled after the National Academy of Engineering Grand Challenges and the Grand Challenges for Social Work, we propose the Grand Challenges for the Age-Friendly Ecosystem, areas in need of action and support, for transformational change in our social, educational, service, and built environments to better meet the needs of our aging society.

